# Specific classification and new therapeutic targets for neuroendocrine prostate cancer: A patient-based, diagnostic study

**DOI:** 10.3389/fgene.2022.955133

**Published:** 2022-09-02

**Authors:** YouZhi Wang, Ning Wu, KeKe Wang, YiHao Liao, JiaNing Guo, BoQiang Zhong, Tao Guo, JiaMing Liang, Ning Jiang

**Affiliations:** ^1^ Department of Urology, Tianjin Institute of Urology, The Second Hospital of Tianjin Medical University, Tianjin, China; ^2^ Key Laboratory of Breast Cancer Prevention and Therapy, State Ministry of Education, National Clinical Research Center for Cancer, Key Laboratory of Cancer Prevention and Therapy, Tianjin Clinical Research Center for Cancer, Tianjin Medical University Cancer Hospital and Institute, Tianjin, China; ^3^ Department of Pathology, The Second Hospital of Tianjin Medical University, Tianjin, China

**Keywords:** neuroendocrine prostate cancer, neuroendocrine differentiation prostate cancer, high-risk prostate cancer, therapeutic targets, metastasis-free survival

## Abstract

**Objective:** Neuroendocrine prostate cancer (NEPC) is an aggressive variant of prostate cancer (PC) that may arise *de novo* or in patients previously treated with hormonal therapies for prostate adenocarcinoma as a mechanism of resistance. In our investigation, there appeared to be a strong correlation between neuroendocrine differentiation prostate cancer (NEDPC) and NEPC. The objectives of this study included exploring whether NEDPC is an intermediate stage in the progression of high-risk prostate cancer (HRPC) to NEPC and identifying risk factors and new targets associated with survival in the treatment of NEPC.

**Methods:** The selected prostate cancer patients were progressed to high-risk and characterized by neuroendocrine. We collected the clinical data and characteristics of patients with three types of cancer: the incidence of metastasis, site and time of metastasis, recurrence rate, related treatment methods, etc. The similarity and differences of the three groups were compared through experiment and database.

**Results:** By analyzing the clinical data and immunohistochemical results, we found that there seems to be a clinical feature of neuroendocrine differentiation (NED) status in between when patients progress from PC to NEPC. Finding novel treatment targets would therefore be beneficial by taking into account NEDPC as the stage of PC progression prior to NEPC. The metastasis-free survival curve and the immunohistochemical results are informing us that NEDPC can be a pre-state for diagnosing NEPC.

**Conclusion:** NEPC is a late PC symptom that is frequently disregarded and has a bad prognosis. Finding novel treatment targets would therefore be beneficial by taking into account NEDPC as the stage of PC progression prior to NEPC.

## Introduction

In Western countries, prostate cancer (PC) has the second highest death rate of all cancer-related diseases, and in recent years, both incidence and mortality rates have been increasing in China (Patel et al., 2019; Siegel et al., 2020). Endocrine medication research and enhanced surgical techniques have proven successful in preventing PC from progressing to an advanced stage. Unfortunately, we still lack cures for advanced castration-resistant prostate cancer (CRPC), which can develop from this disease (Patel et al., 2019). Some studies have regarded NEPC as a subtype of CRPC that is characterized by the absence of PSA, the lack of response to androgen deprivation therapy (ADT), metastasis of visceral organs, and limited prognosis (Wang et al., 2014; Litwin and Tan, 2017; Lee et al., 2019; Patel et al., 2019). Most cases of NEPC occur as a result of ADT or chemotherapies through a process known as therapy-induced neuroendocrine prostate cancers (t-NEPCs) (Beltran et al., 2019). For NEPC derived from adenocarcinoma, Mucci et al. (2000) reported that 10%–100% of patients’ tumor cells also exhibited neuroendocrine differentiation (NED), but only 17%–30% of patients with advanced prostate tumors eventually develop aggressive t-NEPC (Aparicio et al., 2011).

Due to biopsy’s general inability to diagnose advanced diseases, few articles on NEPC progression from conventional PC have been published. Until now, the majority of these cases have been documented in single case reports and small series, which limits our ability to derive definitive conclusions regarding the clinical course, prognosis, or the most effective treatment (Wang et al., 2014; Beltran et al., 2019). As such, we lack an understanding of the intermediate process or stages between PC and NEPC (Mucci et al., 2000). In this study, through statistically analyzing clinical data and pathological diagnosis, we found that NED seems to be an intermediate stage before high-risk prostate cancer (HRPC) progresses to NEPC. Moreover, we compared our analysis of three specific aspects to further investigate the likelihood of NED progression to NEPC.

## Materials and methods

### Patient

**FIGURE 1:**
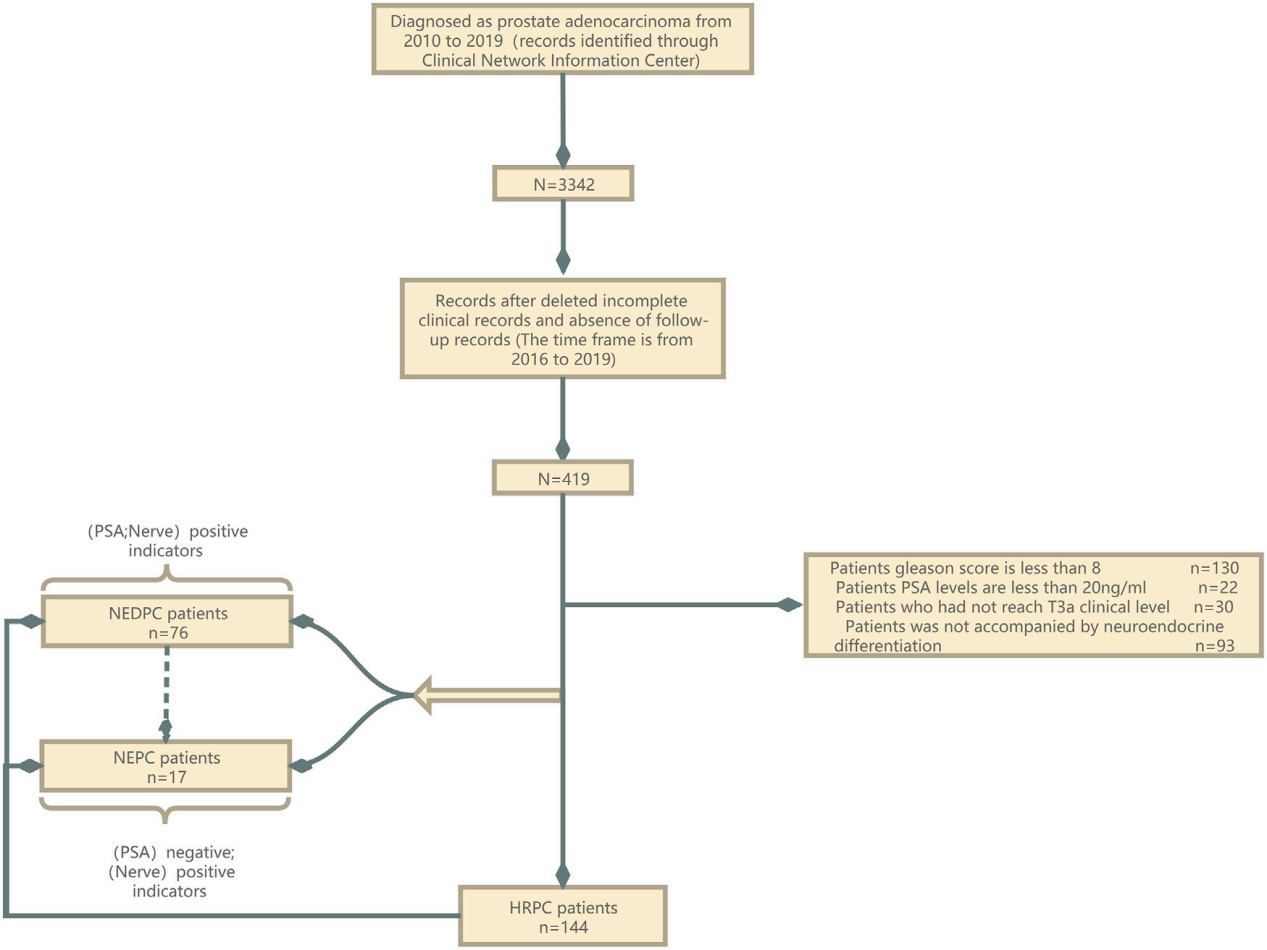
Patients flow diagram. NED, neuroendocrine differentiation; NEPC, neuroendocrine prostate cancer; HRPC, high-risk prostate cancer; PSA, prostate-specific antigen.

A total of 235 PC patients were enrolled in this study after elimination (Figure 1). Between March 2010 and December 2020, we retrospectively reviewed patients who underwent PC therapy including ADT, CT, RT, CRT, and more within our department. There were 7,728 hospitalizations with a total of 3,342 patients recorded. And we concentrated the patients in the final statistics from 2015 to 2019. The following patients were excluded: patients whose Gleason scores were less than 8, patients whose PSA levels were lower than 20 ng/ml, and patients who had not reached the T3a clinical level (Supplementary Box) (Stephenson et al., 2006; Stephenson et al., 2009; Mohler et al., 2016; Nabid et al., 2018; Stelloo et al., 2018). For the 235 patients, we did a study, resected PC samples and LNs were evaluated histopathologically by experienced pathologists. NEDPC and NEPC patients were subdivided according to their pathological diagnoses (PSA positive/negative; Positive neuroendocrine index) and clinical features. NEPC has varied in the previous article, and we need a more standardized definition. Tumor stages were assessed according to the Report of the Advanced Prostate Cancer Consensus Conference APCCC 2017. Subtypes of PC were classified in line with the American Urological Association (AUA)/European Association of Urology (EAU) adenocarcinoma classification (Zelic et al., 2020; Mottet et al., 2021). The predominant pattern was defined as the pattern with the largest percentage.

### Follow-up

Follow-up data were collected through official contact with patients or their relatives by telephone or from hospital records. Each hospitalized patient had complete medical records. We did not include data from patients who were unreachable after surgery in the group that underwent prostate needle biopsy. We compared the patients who had provided follow-up information based on relevant covariances. The result showed that there was no statistically significant difference between the two groups (*p* > 0.5, Data Supplement). Routine examinations such as prostate-specific antigen (PSA) testing, digital rectal examination (DRE), multiparametric magnetic resonance imaging (mpMRI), and CT were commonly performed before surgery. Patients received pathologic diagnosis of prostate cancer about two weeks after prostate needle biopsy. We then performed appropriate surgical procedures according to the diagnoses. Bone scans were performed as clinically indicated on the basis of bone pain in high risk localized prostate cancer with a PSA >20 prior to radical prostatectomy. The primary end point metastasis-free survival (MFS) was calculated as the time interval from the date of surgery to the first event-relapse and metastasis as a result of NEDPC or NEPC-or last follow-up.

### Statistical analysis

All statistical analyses were performed using the IBM SPSS Statistics 25.0 (SPSS, Chicago, IL) software. The associations between the factors, HRPCs, NEDPCs, and NEPCs were evaluated using univariable and multivariable Cox regression models by computing the robust sandwich estimates of the covariance matrix. The hazard ratio (HR) of progress and its 95% CI were calculated for each factor. Metastasis-free survival was estimated using the Kaplan-Meier method and compared using the log-rank test. The quantitative results of immunohistochemistry were observed and analyzed by three experienced pathologists and compared using Imaje J software to avoid false positive results.

### Microarray data analysis

The two public PC microarray gene profiling datasets (GSE33277 and GSE59985) used in our study were downloaded from the Gene Expression Omnibus (GEO) (www.ncbi.nlm.nih.gov/geo). Samples in GSE33277 contained PC, CRPC, and SCC of the prostate, which are considered subtypes of NEPC. Furthermore, GSE33277 also contained data from some xenograft tumors, which may be different from primary tumors in humans. Therefore, we excluded these xenograft samples before data analysis. The dataset of GSE59985 was derived from a unique patient-derived xenograft model of NEPC transdifferentiation. All data filters were processed on the Omincs-Bean Cancer website (www.omicsbeancancer.com). Data quality control was determined using principal component analysis (PCA) on Omincs-Bean Cancer. Gene expression data were analyzed using clustering and correlation heat maps. Differentially expressed genes were identified using a classic *t*-test and *p*-values (*p* < 0.01).

### Gene ontology and pathway enrichment analysis

Gene ontology (GO) is a major type of bioinformatics genetic analysis that unifies the representation of gene and gene product attributes. Biological process analysis was performed using GO and GO Annotations. KEGG is an integrated database resource for the biological interpretation of genome sequences and other high-throughput data. The molecular functions of genes and proteins are associated with ortholog groups and stored in the KEGG Orthology database. Signaling pathways enrichment was analyzed using the KEGG pathway. The protein-protein interaction (PPI) further improved our understanding of the importance of the pathway and kinase proteins. Finally, GO enrichment, KEGG pathway analysis and PPI were performed using Omincs-Bean Cancer online tool. *p* < 0.05 was considered statistically significant.

### Data analyses

Statistical analyses were performed using GraphPad Prism software. The data were presented as mean—SD. A *t*-test was used to analyze the experimental data. *p* < 0.05 was considered statistically significant.

## Results

### Baseline data and metastasis-free survival of neuroendocrine differentiation prostate cancer and neuroendocrine prostate cancer groups

**FIGURE 2:**
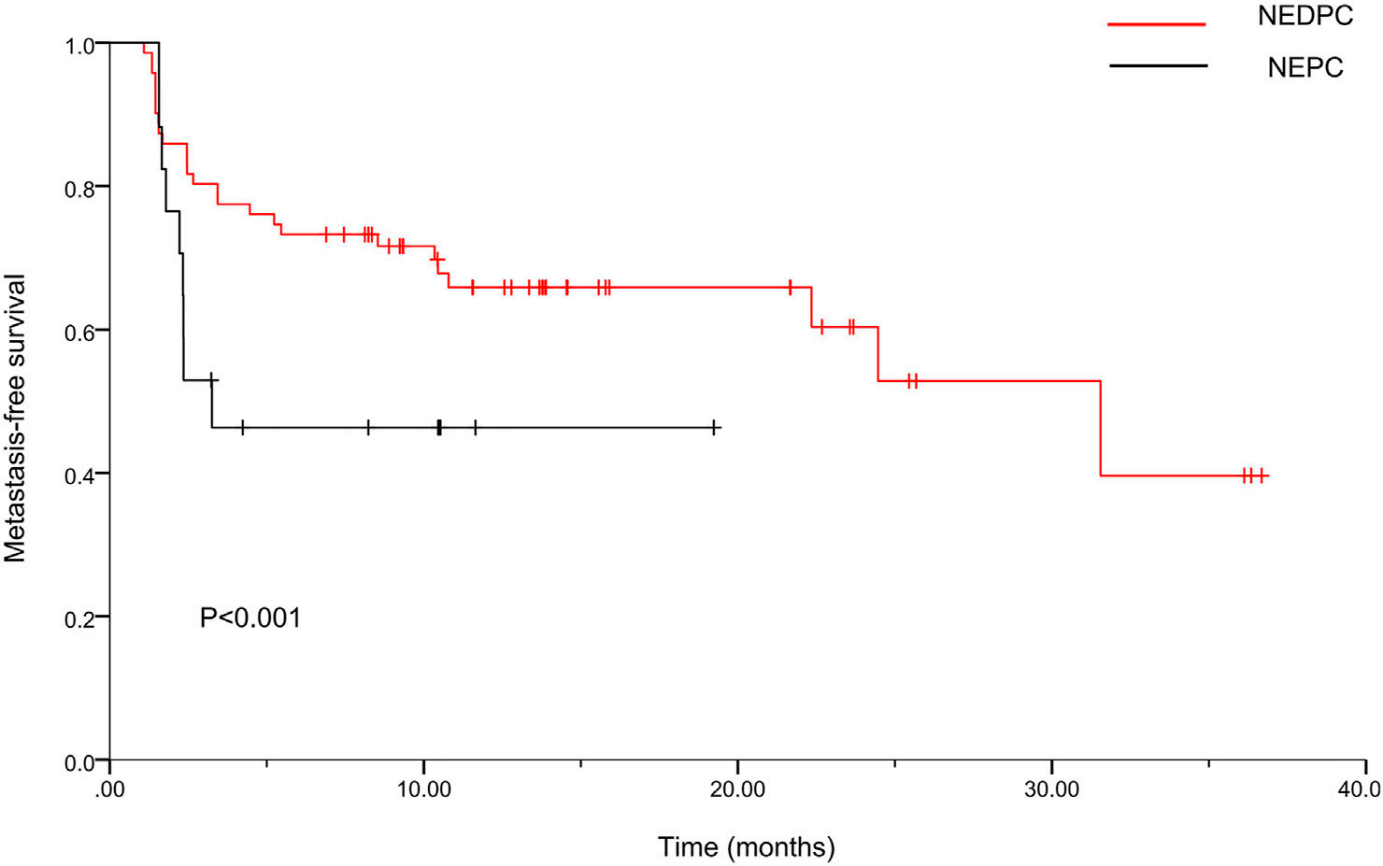
Kaplan-Meier curves for metastasis-free survival (MFS) of two groups. For the NEDPC group, the median MFS was 23.2 months (95% CI, 19.2–27.2). For the NEPC group, the median MFS was 10.1months (95% CI, 5.9–14.2).

Table 1 and Table 2 show the baseline data of all patients (*n* = 235) divided into three groups [HRPC, NEDPC, NEPC]. A total of 144 (61.3%), 74 (31.5%), and 17 (7.2%) patients were assigned to the HRPC, NEDPC, and NEPC groups, respectively. We then made Kaplan-Meier curves for metastasis-free survival (MFS) in both groups. The median MFS was 23.2months (95% CI, 19.2–27.2) in the NEDPC group and 10.1 months (95% CI, 5.9–14.2) in the NEPC group (Figure 2).

### Risk factor analysis for high-risk prostate cancer group

Almost all HRPC patients have undergone surgery and can be roughly divided into the following categories: TPB, TPB + RP, TPB + LRP, TPB + LRP + PLND, and TURP. Among them, 70 patients had undergone TPB surgery, and 65 patients had undergone TPB + RP/LRP surgery (Table 1, Supplementary Figure S1). Some of HRPC patients are treated with ADT (*n* = 26), chemotherapy (*n* = 20), neoadjuvant endocrine therapy (*n* = 5), and radiotherapy (*n* = 6) (Table 1, Supplementary Figure S1). The duration of patients undergoing hormone ablation therapy was shown in Supplementary Figure S1. Influencing factors such as age, smoking index, drinking history, the number and position of metastasis, treatment, PSA, fPSA, F/T (%), AR, CgA, CD56, Syn, clinical stage/T, and Gleason score were shown in (Table 3, Supplementary Figure S1). Further univariate and multivariate logistic analyses were performed for factors that were statistically significant. Result indicated that univariable logistic analysis bone metastasis (*p* < 0.001), bladder metastasis (*p* = 0.03), metastatic organs (≥2 *v* < 2; *p* = 0.003), type of Treatment (CT *v* surgery; *p* = 0.003, CRT *v* surgery; *p* = 0.003), multivariate logistic analysis bone metastasis (*p* < 0.001), bladder metastasis (*p* = 0.007) and metastatic organs (≥2 *v* < 2; *p* = 0.01).

### Comparison of the neuroendocrine differentiation prostate cancer and neuroendocrine prostate cancer groups by analyzing metastasis-free survival factors

Both NEDPC and NEPC patients were treated with surgery, which can be roughly divided into the following categories: TPB, TPB + RP, TPB + LRP, TPB + LRP + PLND and TURP. In NEDPC group, 17 patients underwent TPB surgery, and 34 patients underwent TPB + RP/LRP surgery (Table 2, Supplementary Figure S2). Part of NEDPC patients were treated with ADT (*n* = 19), chemotherapy (*n* = 14), neoadjuvant endocrine therapy (*n* = 2), and radiotherapy (*n* = 8) (Table 2, Supplementary Figure S2). Among NEPC group, 13 patients underwent TPB surgery, and 1 patients underwent TPB + RP surgery (Table 2, Supplementary Figure S3). Part of NEPC patients were treated with ADT (*n* = 6), chemotherapy (*n* = 6), neoadjuvant endocrine therapy (*n* = 2), and radiotherapy (*n* = 3) (Table 2, Supplementary Figure S2). The duration of patients undergoing hormone ablation therapy was shown in Supplementary Figures S2, S3. Several variables, including bone metastasis, liver metastasis, bladder metastasis, metastatic organs, and type of NEDPC treatment, were all significant factors for MFS according to univariate analysis (*p* < 0.001, *p* = 0.04, *p* = 0.03, ≥2 *v* < 2; *p* = 0.004, and CT *v* surgery; RT *v* surgery; CRT *v* surgery; *p* = 0.02; *p* < 0.001; *p* = 0.002, respectively) (Table 4).

## Immunohistochemical index and cell morphology of neuroendocrine differentiation prostate cancer and neuroendocrine prostate cancer

**FIGURE 3:**
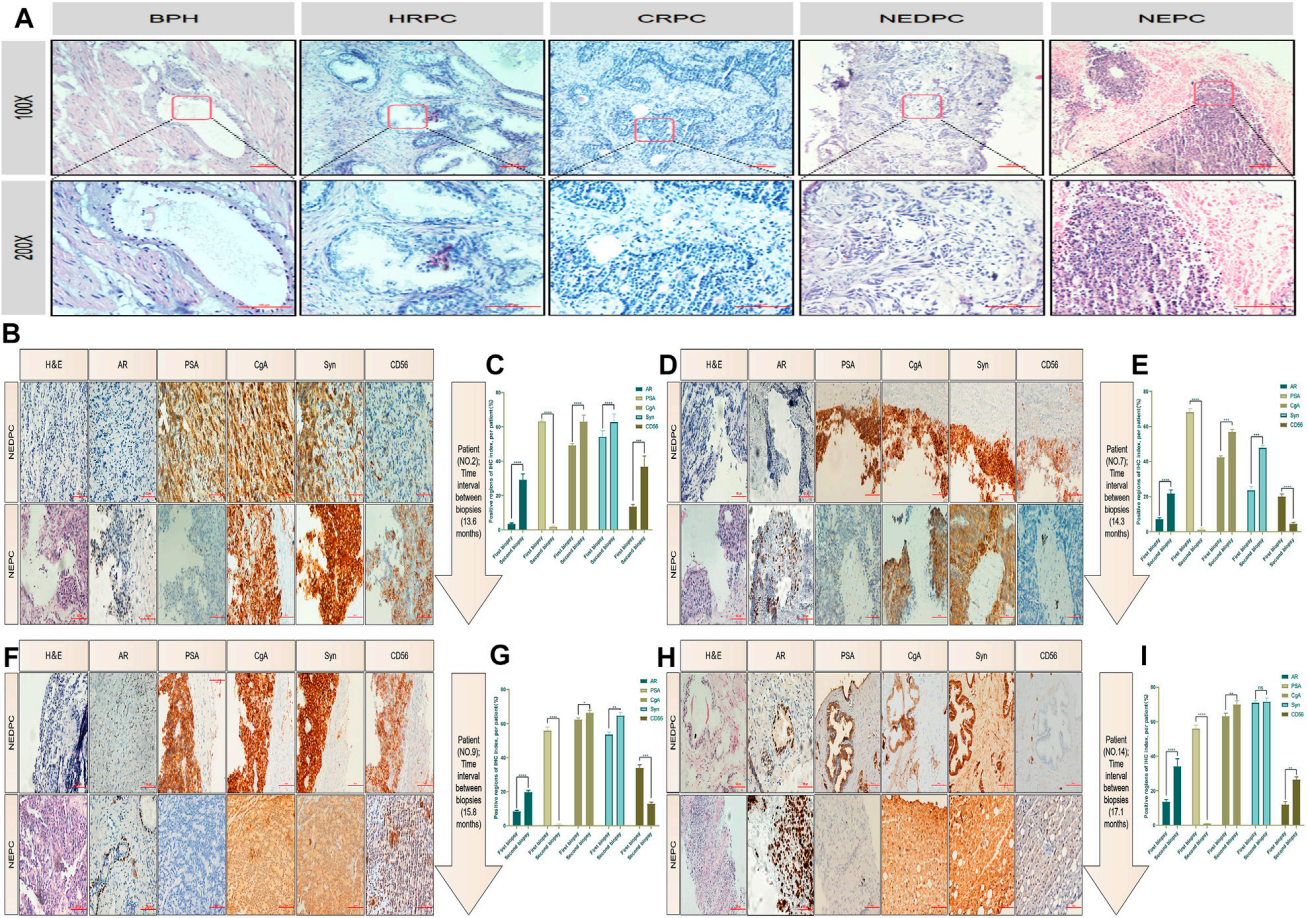
HE staining and immunohistochemical staining for patients. **(A)** Malignant degree of prostate tissue of HE staining, including BPH, HRPC, CRPC, NEDPC, NEPC. **(B)** HE staining and Immunohistochemical staining of the patient’s (Numble 2) two prostate biopsy tissues, including AR, PSA, CgA, Syn and CD56 indicators. **(C)** Comparison of quantitative results of four indicators of prostate tissues immunohistochemistry staining at an interval of 13.6 months (Patient No. 2). **(D)** HE staining and Immunohistochemical staining of the patient’s (Numble 7) two prostate biopsy tissues, including AR, PSA, CgA, Syn and CD56 indicators. **(E)** Comparison of quantitative results of four indicators of prostate tissues immunohistochemistry staining at an interval of 14.3 months (Patient No. 7). **(F)** HE staining and Immunohistochemical staining of the patient’s (Numble 9) two prostate biopsy tissues, including AR, PSA, CgA, Syn and CD56 indicators. **(G)** Comparison of quantitative results of four indicators of prostate tissues immunohistochemistry staining at an interval of 15.8 months (Patient No. 9). **(H)** HE staining and Immunohistochemical staining of the patient’s (Numble 14) two biopsy tissues, including AR, PSA, CgA, Syn and CD56 indicators. The first biopsy site was the prostate and the second was the liver. **(I)** Comparison of quantitative results of four indicators of prostate and liver tissues immunohistochemistry staining at an interval of 17.1 months (Patient No. 14).

To obtain a clearer understanding of the pathologic patterns of PC progression, we first compared the pathological morphology and malignant degree of different types of PC using H & E staining. We included patients of benign prostatic hyperplasia (BPH), HRPC, castration-resistant prostate cancer (CRPC), NEDPC and NEPC (Figure 3A). Next, the clinical and pathology data were respectively summarized in detail (Tables 1, 2). We then performed immunohistochemistry (IHC) on NEPC patients to examine if there was a dynamic change from NEDPC to NEPC. The patients were treated with ADT and underwent tissue biopsy twice, respectively. Patient No. 2 was treated with Bicalutamide + Triptorelin (2 years and 2 months), Abiraterone + prednisone (2 years), and carboplatin (4 months). Patient No. 7 was treated with Bicalutamide + leuprorelin (2 years and 3 months), and etoposide + cisplatin (6 months). Patient No. 9 was treated with Bicalutamide + leuprorelin (4 years and 7 months), and abiraterone + prednisone (5 years). Patient No. 14 was treated with Bicalutamide + leuprorelin, and docetaxel (6 months). Patients (No. 2, No. 7, No. 9) received prostate biopsy for both operations, (interval of time was 13.6; 14.3; 15.8 months). Their pathology reports showed positive PSA for the first biopsy and negative for the second biopsy. The first biopsy for patient No. 14 was prostatic biopsy and the second was liver tissue (highly considered PC metastasis) with an interval of 17.1 months. Therefore, immunohistochemical staining was performed on the biopsy tissues of patients with different indicators, and results were compared and analyzed in detail (Figures 3B,D,F,H). In the process of immunohistochemical results quantification (Figures 3C,E,G,I), we observed significant positive degrees of neuroendocrine indicators at two different stages, and the cell morphology of the biopsy tissues of the patients showed significant progression in the direction of small cell carcinoma. In NEDPC, the location of the PSA positive region and the expression of neuroendocrine indicators in the corresponding location of the patient were also positive, and PSA expression was positively correlated with neuroendocrine indicators expression. It is difficult to identify whether drug treatment or an unpredictable factor led to progression towards NEPC. However, we are confident that patients did exhibit a NEDPC to NEPC transition process during a time interval typically over a year.

## Sets of protein kinases expression show a reversal during neuroendocrine prostate cancer transdifferentiation

**FIGURE 4:**
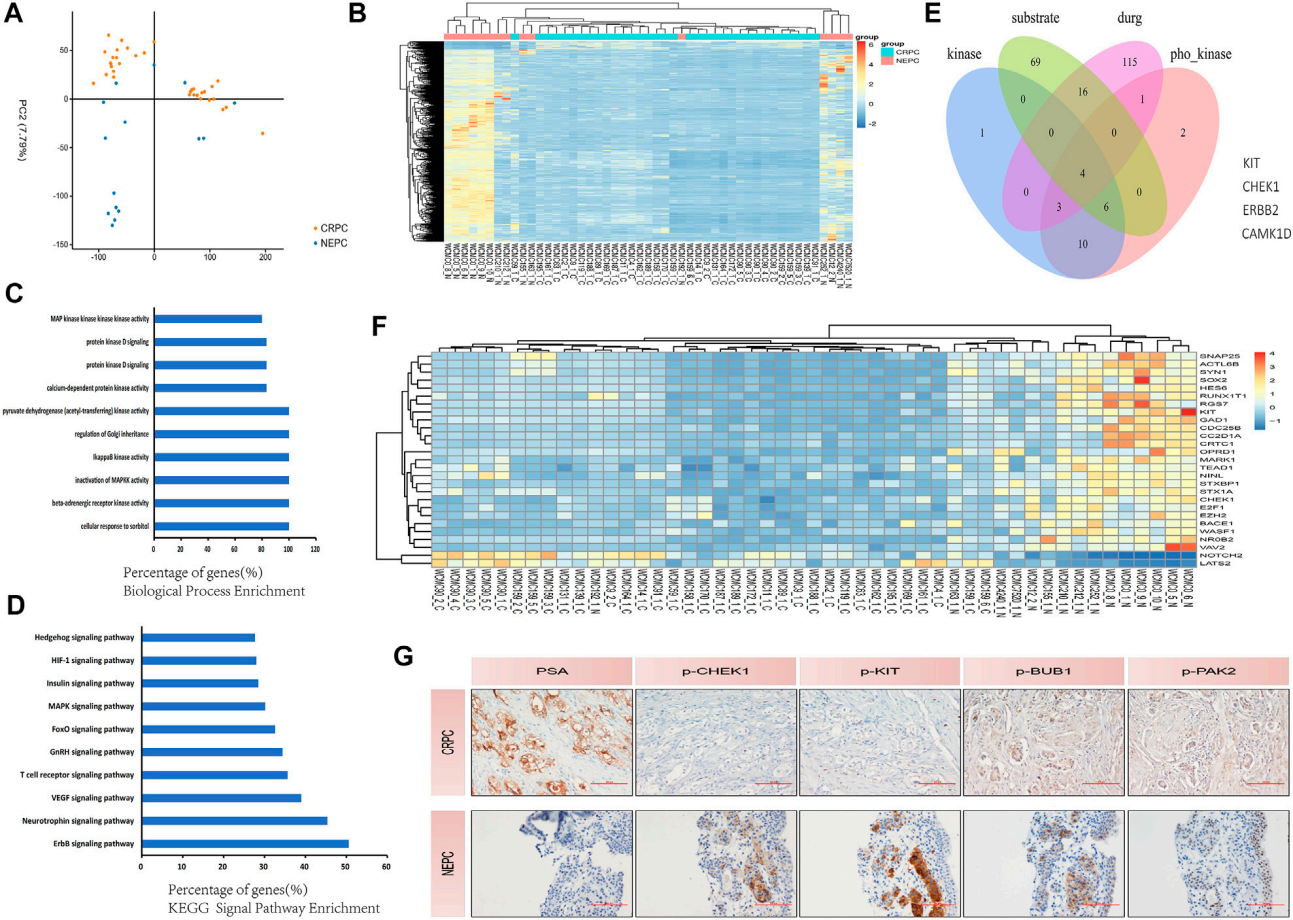
Protein kinases are involved in the development of NEPC. **(A)** PCA plot separated the PC tissues using expression data clustered into CRPC and NEPC (SCC of prostate). The blue, brown, and plots represent NEPC (SCC of prostate) and CRPC, respectively. **(B)** Hierarchical clustering of protein kinase differential expression profifiles among the CRPC group and NEPC (SCC of prostate) group in 49 samples. The heat maps are based on expression values of signifificantly differentially expressed protein kinases (*p* < 0.05) detected by microarray probes. ‘‘Red’’ and ‘‘Blue’’ indicate expression above and below, respectively, relative expression. **(C)** Biological process enrichment of the protein kinases. In biological process enrichment, protein kinases were primarily associated with the phosphatidylinositol metabolic process, signal transduction, the protein modification process, and protein phosphorylation. **(D)** KEGG pathway enrichment analyses demonstrate the signifificance of protein kinases. Protein kinases were mainly associated with several signaling pathways that could affect PC progression in the analysis of KEGG (*p* < 0.01). **(E)** Selected protein kinases with increasing expression, which were associated with phosphorylated kinase substrate and targeted drugs as indicated by Venn diagrams: four protein kinases were selected. **(F)** The heat map shows the increasing expression during NEPC transdifferentiation of the 27 selected phosphorylated and drug-targeted protein kinases. ‘‘Red’’ and ‘‘Blue’’ indicate expression above and below, respectively, relative expression. The heat maps are based on expression values of signifificantly differentially expressed protein kinases (*p* < 0.05) detected by microarray probes. **(G)** Immunohistochemical staining of the CRPC patient’s prostate tissue and the NEPC patient’s prostate tissue, including PSA, p-CHEK1, p-KIT, p-BUB1 and p-PAK2 indicators. NEPC, neuroendocrine prostate cancer; CRPC, castration-resistant prostate cancer; KEGG, Kyoto Encyclopedia of Genes and Genomes pathway; PCA, principle component analysis; PC, prostate cancer; SCC, small cell carcinoma.

At present, the number of patients with NEPC is still increasing makes finding corresponding therapeutic targets an urgent matter. A dataset of GSE33277 containing several samples from these two different pathological types of PC [CRPC and NEPC (SCC of the prostate)] was used in our preliminary study. We used PCA to accurately sort 49 samples based on the two different pathological types of PC (Figure 4A) before conducting bioinformatics analysis. Next, we analyzed the differential expression of protein kinases (PKs) among these two groups of samples. Hierarchical clustering revealed that both CRPC and NEPC (SCC of the prostate) clustered independently from the primary PC (Figure 4B). To develop a basic understanding of the biological processes and pathways enriched in the development of NEPC, we performed GO and pathway enrichment analyses for the differentially expressed PK’s. Analysis indicated that these differently expressed PK’s were primarily involved in the phosphatidylinositol metabolic process, signal transduction, the protein modification process, protein phosphorylation and more (Figure 4C). KEGG signal pathway analysis revealed several signaling pathways that were enriched in CRPC and NEPC (SCC of the prostate), including the VEGF signaling pathway, GnRH signaling pathway and mTOR signaling pathway (Figure 4D). Furthermore, we found 24 PK’s for the above classes respectively and performed deeper screening based on phosphorylated kinase substrate and druggable targets levels (Figure 4E). Finally, we identified 27 actionable and druggable targets among these drastically changed PK’s during NEPC transdifferentiation (Figure 4F). These findings suggest that the expression of a range of proteins is reversed during NEPC transdifferentiation, and some of them may have a critical impact on this process. In addition, we performed immunohistochemistry (IHC) on CRPC patients and NEPC patients to compare their different levels of phosphorylated kinase expression (Figure 4G).

## Discussion

**FIGURE 5:**
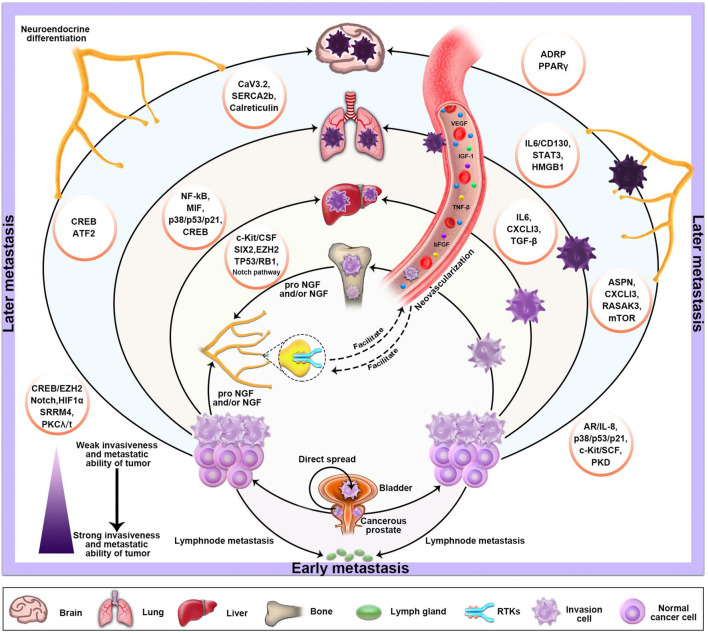
The basic metastatic sites and pathways of PC and the factors which influence its NED and neovascularization. The progression of PC depends on stimulation of the autonomic nervous system. Sympathetic nerve releases norepinephrine (NA) in the tumor microenvironment, activating the adrenergic nerve signal, which is necessary in the early stages of tumor growth and angiogenesis. Moreover, the various molecular mechanisms that primarily mediate NED are also described in the diagram.

The origin of NEPC is still controversial. Some scholars believe that NEPC is related to APUD cells and tumor stem cells in the prostate, while others believe that it originates from basal cells. In addition, some studies have found that NEPC can be differentiated from the luminal epithelial cells of the prostate by mice tumor models. However, some scholars and studies have shown that NEPC can also occur *de novo*, which means that prostate cancer patients are first diagnosed with NEPC. The definition of neuroendocrine is mainly the positive of neuroendocrine indexes (CgA, CD56, Syn). Despite the current controversy, pathological diagnosis is still the gold standard for the diagnosis of NEPC. Therefore, we base our assessment of the neuroendocrine diagnosis on the observation of pathologists after surgery. Pure small cell carcinoma represents a subset of NEPC, which is defined according to the histopathologic features discussed. NEPC in turn represents a subset of a broader clinically defined PC phenotype that displays relative resistance to androgen receptor signaling inhibition. As for NEDPC, we prefer to define it as PC progressing to an intermediate state of NEPC. It is mainly a mixture of small cell carcinoma and adenocarcinoma and also expresses neuroendocrine markers, but has a lower expression of AR and a higher expression of PSA compared to NEPC. Finally, the two aggregated clinical datasets were compared to HRPC patients. The fundamental goal of this study was to provide clinical doctors and pathologists with a better understanding of NEPC. Our main finding was a new association between NEDPC and NEPC, for both of which we calculated the median MFS [MFS was 23.2 months (95% CI, 19.2–27.2) for the NEDPC group and 10.1 months (95% CI, 5.9–14.2) for the NEPC group]. By paying vigilant attention to NEDPC and NEPC correlation, doctors can draft more precise plans for the further treatment of patients. Opinions on the origin of NEPC are non-unanimous; various theories include primary small-cell NEPC, tNEPC [a late manifestation of metastatic castration-resistant prostate cancer (mCRPC)] and more. Clinical criteria for the recognition of the NEPC phenotype have been proposed and generally accepted. Different criteria proposed by different clinicians/researchers groups share many common features (Beltran et al., 2014; Tagawa, 2014; Berchuck et al., 2021). Due to the uncertainty of the source of NEPC, its specific classification or diagnostic criteria remain undetermined in many aspects. Patients diagnosed with NEPC are usually treated with palliative treatment (PT) and chemotherapy (CT) because establishing an optimal treatment strategy has been difficult (Attard et al., 2016). Moreover, numerous studies have found that the late stages of some malignant tumors or metastatic tumors (prostate cancer, lung cancer, gastrointestinal cancer) are often accompanied by neuroendocrine differentiation and neovascularization (Tagawa, 2014). NED in cancer tissues generally refers to the occurrence of NED in some tumor cells, which differs from most endocrine gland tumors and neuroendocrine tumors (Attard et al., 2016). These Ned-like tumor cells are a concomitant component of tumor tissue and part of cancer tissues. NEPC is a poorly differentiated small cell carcinoma and a highly angiogenic tumor (Attard et al., 2016). Like many advanced neuroendocrine tumors, it has aggressive and resistant qualities and distinct cellular morphology. Information regarding the specific links between angiogenesis and the NED of NEPC is not sufficient (Li et al., 2017; Li et al., 2019; Zhao et al., 2019). So what roles do NED and angiogenesis play in the progression from NEDPC to NEPC? The obvious increase in metastatic sites and the degree of metastatic malignancy suggest that the interaction between tumor neovascularization and NED is crucial (Chen et al., 2018; Puca et al., 2019; Teo et al., 2019). Therefore, in the progression from NEDPC to NEPC, we envisage that early angiogenesis is closely related to neuroendocrine differentiation and will continue to facilitate neurodifferentiation. Moreover, we also predict that we can inhibit NEPC progression by inhibiting angiogenesis as a new therapeutic target (Figure 5). Therapeutic, molecular, and cellular factors involved in the regulation of NED in cancer tissues are diverse. Various factors affecting PC cells include androgen deprivation, radiation, and chemotherapy (Haberkorn et al., 2016; Teo et al., 2019; Wang et al., 2021). In addition, tumor microenvironmental cells (TME) including mast cells, tumor-associated fibroblasts (CAFs), macrophages, and bone marrow stromal cells (BMSCs) have been shown to promote NED (Chan et al., 2017; Raphael et al., 2017; Ramnarine et al., 2018; Rooper et al., 2018; Su et al., 2018; Zhang et al., 2018).

So we assumed that there was also a process between PC and NEPC. What methods can we use to clarify this process and how can we prevent NE like differentiation of prostate cancer? To investigate, we collected clinical information and immunohistochemical validation. As established, NEPC was distinguished from prostate adenocarcinoma based on NE markers (NSE, CgA, Syn, CD56) and the loss of PSA expression. In our study, 76% of NEPCs were CgA positive and 88% were Syn positive, but only 6% of NEPCs were PSA positive. On the other hand, 70% and 95% of NEDPC were CgA, Syn positive but 89% were PSA positive. It is worth noting that we found both NE markers (CgA, Syn, and CD56) and positive PSA in NEDPC by IHC. However, PSA became negative and NE markers grew stronger when NEDPC progressed into NEPC. Our data supported that NEDPC cells derived from prostate adenocarcinoma epithelial cells and trans-differentiated into NE-like cells. This was consistent with Vashchenko’s report that the origin of malignant NE-like cells should be luminal cells based on their double-positive staining of luminal markers and NE markers (Lee et al., 2018). Our data suggested the discrimination diagnosis between NEDPC and NEPC was not only based on histologic morphology, but IHC of PSA and NE markers also played important roles.

From a total of 17 patients with NEPC, we observed that frequent metastases, low PSA expression, and small cell morphology were consistent with existing research (Lee et al., 2018). Moreover, the median MFS of NEDPC and NEPC were 23.2 and 10.1 months respectively. This indicated that the emergence of NED posed significant clinical challenges as the survival rates decreased and NEPC survival rates were also extremely poor, predicting the pre-stage of NEPC development may provide a rationale for early intervention and treatment for the NEDPC stage. Our analysis of influencing risk factors for HRPC such as age, smoking index, drinking history, number and position of metastasis, treatment, and Gleason score are shown in Table 3. Our data (bone metastasis (*p* < 0.001), bladder metastasis (*p* = 0.03), metastatic organs (≥2 *v* < 2; *p* = 0.003)) suggested HRPC patients are at increased risk for micrometastatic disease and it is reasonable to employ a more aggressive treatment plan for HRPC patients.

Furthermore, important prognostic factors for NEPCs include high aggressiveness and organ metastasis. Our study found that patients with bone metastasis had higher survival rates than those with lung, liver, brain, bladder, or other organ metastases or those without them. This suggests that organs with metastasis may reflect a biological progression of NED cells. Papandreou et al. (2002) reported that the number of organs with metastasis involved was an independent and a poor prognostic factor for survival after NEPC (Papandreou et al., 2002; Vashchenko and Abrahamsson, 2005; Zhang and Hao, 2021) (HR, 2.21; 95% CI, 1.01–4.87).

Our study has several limitations. First of all, our research was a single-center retrospective study even though PSW was utilized to balance the factors that might affect results between the groups. Secondly, the number of patients with NEDPC and NEPC is small, which may have increased the possibility of selection bias. Third, due to various reasons such as the physical discomfort of the patients, we were unable to conduct multiple intermittent biopsies for all 17 NEPC patients, which also impacted our experimental results to an extent. Lastly, we only compared the NEDPC and NEPC statistics and biopsies and have not been able to further identify the processes between the NEDPC and NEPC signaling pathways to describe the connections between the two. We can provide a list of factors that may influence NED’s further progress in Figure 5. However, it is extremely important to classify NEPC, and when a patient’s pathological diagnosis and clinical manifestations indicate the NEDPC stage, we must closely observe the dynamics of the patient, consider biopsy in months and observe the progress of the organs at the site of metastasis. Additionally, the therapeutic targets we screened were further confirmed by patients and cell lines, and the outcomes were as we anticipated, allowing for novel therapeutic avenues for NEPC. Future research would be required to more precisely pinpoint the illness categories or therapies that might be connected to NEPC. In conclusion, we prefer to define NEDPC as PC progressing to an intermediate state of NEPC. It is mainly a mixture of small cell carcinoma and adenocarcinoma and also expresses neuroendocrine markers, but has a lower expression of AR and a higher expression of PSA compared to NEPC. It would be valuable to consider NEDPC as a state of PC progression prior to NEPC, which provides clinicians with a new diagnostic criterion and helps create better therapeutic techniques for patients. Once NEDPC is diagnosed, NEDPC patients need to undergo repeated punctures to observe changes in immunohistochemical markers (PSA and AR), and a new review of their serum PSA is required to observe the progression of their disease. More importantly, the type of treatment and number of metastatic organs are the most important factors related to surviving the progression to NEPC. In addition, it is particularly important to find NEPC’s key targets in the case of endocrine therapy failure. Our study found that PSA and NE markers were the main factors contributing to the diagnosis for both NEPC and NEDPC. Furthermore, the four novel phosphorylated kinases (KIT, CHEK1, BUB1, PAK2) provide a new direction for clinical therapy.

## Data Availability

The original contributions presented in the study are included in the article/Supplementary Material, further inquiries can be directed to the corresponding author.
