# Supplementary figures and images for: Specific classification and new therapeutic targets for neuroendocrine prostate cancer: A patient-based, diagnostic study
Source: Front Genet. 2022 Sep 2;13:955133. doi: 10.3389/fgene.2022.955133 (PMC9479159; doi:10.3389/fgene.2022.955133)

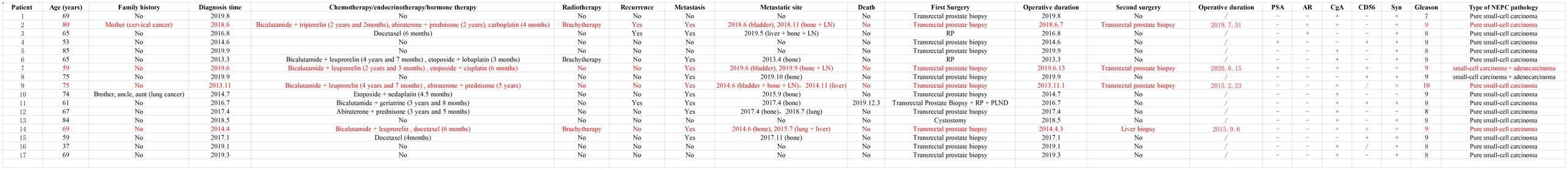

Supplement: Supplementary file 1 [file Image3.JPEG]

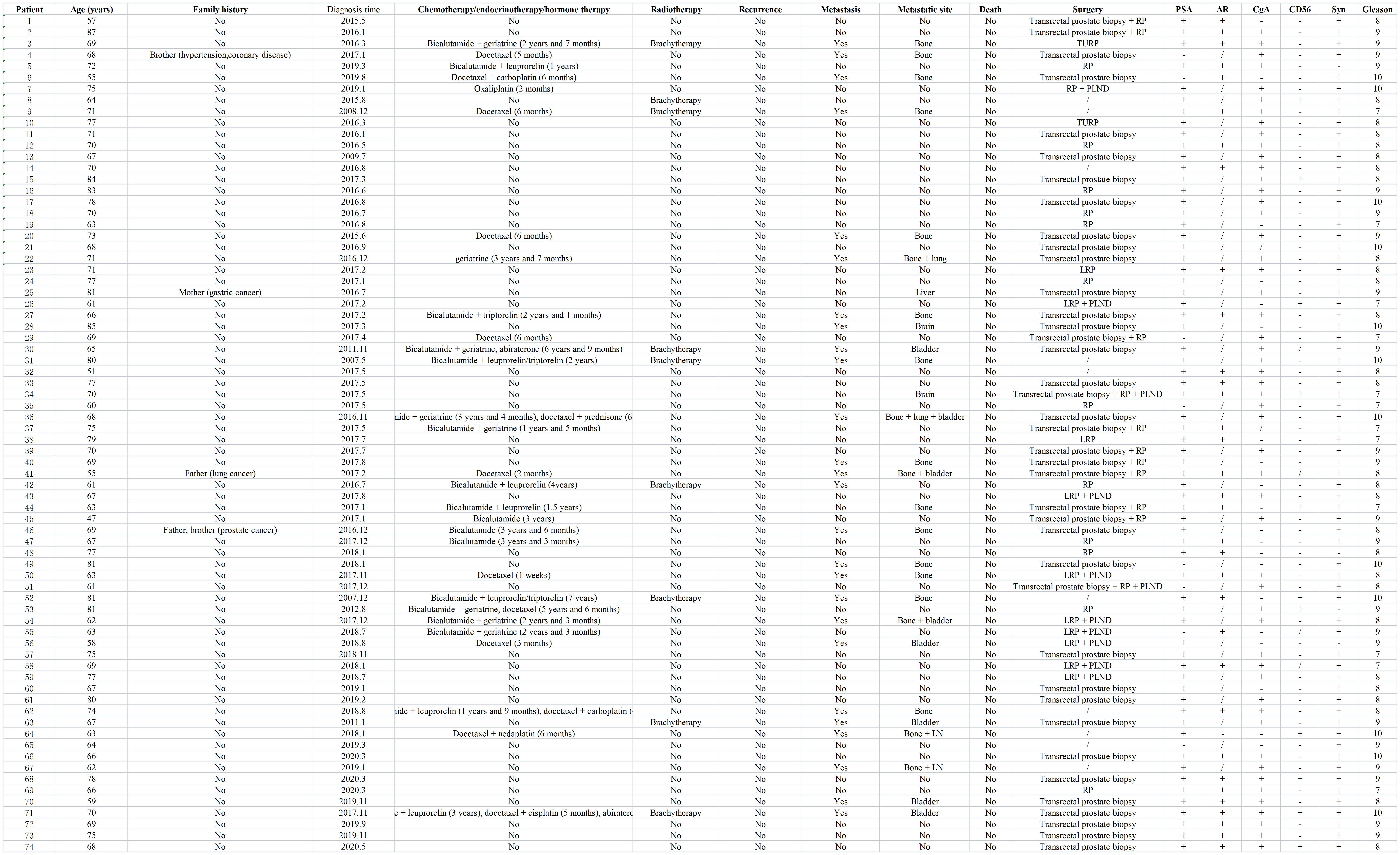

Supplement: Supplementary file 4 [file Image2.JPEG]
