# Supplementary material for: Specific classification and new therapeutic targets for neuroendocrine prostate cancer: A patient-based, diagnostic study
Source: Front Genet. 2022 Sep 2;13:955133. doi: 10.3389/fgene.2022.955133 (PMC9479159; doi:10.3389/fgene.2022.955133)
Supplement: Supplementary file 2 [file Table1.docx]

| Table1. Patient Characteristics at HRPC Diagnosis and Treatment | | |
| --- | --- | --- |
| Characteristic No. of Patients % | | |
| Age, years | | |
| Median | 69.5 |  |
| Range | 50-89 |  |
| Induction factors | | |
| Smoking index | | |
| <200 | 7 | 13.2 |
| 200-400 | 5 | 9.4 |
| >400 | 41 | 77.4 |
| Drinking | | |
| Yes | 27 | 18.8 |
| No | 117 | 81.2 |
| Clinical stage | | |
| No-metastasis | 118 | 81.9 |
| Metastasis | 26 | 18.1 |
| Gleason score | | |
| 8 | 89 | 61.8 |
| 9 | 47 | 32.6 |
| 10 | 8 | 5.6 |
| Types of drugs (chemotherapy; endocrinotherapy; hormone therapy) | | |
| Bicalutamide/+geriatrine/leuprorelin | 26 | 53.1 |
| Docetaxel/+bicalutamide/carboplatin/nedaplatin | 18 | 36.7 |
| Neoadjuvant endocrine therapy | 5 | 10.2 |
| Types of treatment | | |
| RT | 6 | 3.7 |
| CRT | 3 | 1.8 |
| CT | 20 | 12.1 |
| Surgery | | |
| 【TPB】 | 70 | 42.7 |
| 【TPB with LRP/RP】 | 65 | 39.6 |
| Abbreviations: HRPC, high-risk prostate cancer; RT, radiotherapy; CRT, chemoradiotherapy; CT, chemotherapy; TPB, template biopsy of the prostate; LRP, laparoscopic radical prostatectomy; RP, radical prostatectomy. | | |
