# Supplementary material for: Specific classification and new therapeutic targets for neuroendocrine prostate cancer: A patient-based, diagnostic study
Source: Front Genet. 2022 Sep 2;13:955133. doi: 10.3389/fgene.2022.955133 (PMC9479159; doi:10.3389/fgene.2022.955133)
Supplement: Supplementary file 5 [file Table2.docx]

| Table2. Patient Characteristics at NEPC/NEDPC Diagnosis and Treatment | | |
| --- | --- | --- |
| Characteristic No. of Patients % | | |
| Age, years | | |
| Median | 69*/69^#^ |  |
| Range | 37-85*/47-87^#^ |  |
| Type of NEPC pathology | | |
| Pure SCC | 15* | 88.2* |
| Adeno-SCC | 2* | 11.8* |
| LCNEPC | 0* | 0* |
| PSA | | |
| Negative | 16*/8^#^ | 94.1*/10.8^#^ |
| Positive | 1*/66^#^ | 5.9*/89.2^#^ |
| Main sites of metastasis | | |
| Bone | 11*/20^#^ | 37.9*/48.8^#^ |
| Lung | 2*/2^#^ | 6.9*/4.9^#^ |
| Liver | 3*/1^#^ | 10.3*/2.4^#^ |
| LN | 4*/2^#^ | 13.8*/4.9^#^ |
| Bladder | 3*/8^#^ | 10.3*/19.5^#^ |
| Brain | 0*/2^#^ | 0*/4.9^#^ |
| Two or more metastasis | 6*/6^#^ | 20.7*/14.6^#^ |
| Immunohistochemical index[CgA] | | |
| Negative | 4*/20^#^ | 23.5*/27.8^#^ |
| Positive | 13*/52^#^ | 76.5*/72.2^#^ |
| Immunohistochemical index[Syn] | | |
| Negative | 2*/4^#^ | 11.8*/5.4^#^ |
| Positive | 15*/70^#^ | 88.2*/94.6^#^ |
| Types of drugs (chemotherapy; endocrinotherapy; hormone therapy) | | |
| Bicalutamide/+Geriatrine/Leuprorelin | 6*/19^#^ | 42.9*/55.9^#^ |
| Docetaxel/+Carboplatin/Nedaplatin | 3*/13^#^ | 21.4*/38.2^#^ |
| Etoposide/+Platinum | 3*/0^#^ | 21.4*/0^#^ |
| Abiraterone/+Platinum | 2*/2^#^ | 14.3*/5.9^#^ |
| Types of treatment | | |
| PT | 7*/21^#^ | 20.6*/20.6^#^ |
| RT | 3*/9^#^ | 8.8*/8.8^#^ |
| CRT | 3*/7^#^ | 8.8*/6.9^#^ |
| CT | 7*/14^#^ | 20.6*/13.7^#^ |
| Surgery |  |  |
| 【TPB】 | 13*/17^#^ | 38.2*/16.7^#^ |
| 【TPB with RP】 | 1*/34^#^ | 2.9*/33.3^#^ |
| Abbreviations: *, NEPC; ^#^,NED; NEPC, neuroendocrine prostate cancer; NEDPC, neuroendocrine differentiation prostate cancer; Pure SCC, pure small-cell carcinoma; Adeno-SCC, SCC admixed with adenocarcinoma; LCNEPC, large-cell NEPC; PSA, prostate-specific antigen; LN, lymph node; CgA, chromogranin A; Syn, synaptophysin; RT, radiotherapy; CRT, chemoradiotherapy; CT, chemotherapy; TPB, template biopsy of the prostate; RP, Radical prostatectomy. | | |
