# Supplementary material for: Specific classification and new therapeutic targets for neuroendocrine prostate cancer: A patient-based, diagnostic study
Source: Front Genet. 2022 Sep 2;13:955133. doi: 10.3389/fgene.2022.955133 (PMC9479159; doi:10.3389/fgene.2022.955133)
Supplement: Supplementary file 6 [file Table3.docx]

| Table3. Patient and Tumor Characteristics at HRPC Diagnosis | | | | |
| --- | --- | --- | --- | --- |
| Covariate | Level | Hazard Ratio | 95% CI | P |
| Univariable Cox model | | | | |
| Age at HRPC diagnosis | Continuous | | | |
| Age at HRPC diagnosis | ≤69 *v* ＞69 | 1.12 | 0.51-2.45 | 0.77 |
| Smoking index | ≥200 *v* ＜200 | 1.43 | 0.64-3.21 | 0.39 |
| Drinking | Yes *v* no | 1.36 | 0.51-3.65 | 0.54 |
| Bone metastasis | Yes *v* no | 21.25 | 8.50-53.11 | ＜0.001 |
| Liver metastasis | Yes *v* no | 2.41 | 0.31-18.45 | 0.40 |
| Bladder metastasis | Yes *v* no | 4.05 | 1.18-13.92 | 0.03 |
| Metastatic organs | ≥2 *v* ＜2 | 6.62 | 1.93-22.79 | 0.003 |
| Stage at PCa diagnosis | Metastasis *v* nonmetastasis | 2.61 | 0.90-7.57 | 0.08 |
| Gleason score | ≤9 *v ＞*9 | 0.38 | 0.09-1.64 | 0.20 |
| Type of Treatment | CT *v* surgery  RT *v* surgery  CRT *v* surgery | 5.46  5.86  10.24 | 1.79-16.66  0.64-53.46  2.45-46.62 | 0.003  0.12  0.003 |
| Multivariable Cox model | | | | |
| Bone metastasis | Yes *v* no | 29.62 | 5.27-166.51 | ＜0.001 |
| Bladder metastasis | Yes *v* no | 20.98 | 2.33-188.75 | 0.007 |
| Metastatic organs | ≥2 *v* ＜2 | 12.25 | 1.81-82.90 | 0.01 |
| Abbreviations: HRPC, high-risk prostate cancer; RT, radiotherapy; CRT, chemoradiotherapy; CT, chemotherapy. | | | | |
