# Supplementary material for: Specific classification and new therapeutic targets for neuroendocrine prostate cancer: A patient-based, diagnostic study
Source: Front Genet. 2022 Sep 2;13:955133. doi: 10.3389/fgene.2022.955133 (PMC9479159; doi:10.3389/fgene.2022.955133)
Supplement: Supplementary file 7 [file Table4.docx]

| Table4. Survival After NEPC/NEDPC Diagnosis by Univariable and Multivariable Cox Models by the Robust Sandwich Estimates of the Covariance Matrix | | | | | |
| --- | --- | --- | --- | --- | --- |
| Group | | NEPC | | NEDPC | |
| Covariate | Level | Hazard Ratio  (95% CI) | P | Hazard Ratio  (95%CI) | P |
| Univariable Cox model | | | | | |
| Age at NEPC/NEDPC diagnosis | Continuous | | | | |
| Age at NEPC/NEDPC diagnosis | ≤69 *v* ＞69 | 1.10(0.28-4.28) | 0.90 | 1.74( 0.87-3.49) | 0.12 |
| Bone metastasis | Yes *v* no | 10.83(1.36-86.00) | 0.02 | 5.27(2.39-11.62) | ＜0.001 |
| Lung metastasis | Yes *v* no | 1.20(0.25-5.79) | 0.82 | 2.21( 0.67-7.34) | 0.19 |
| Liver metastasis | Yes *v* no | 2.31(0.58-9.27) | 0.24 | 3.59(1.08-11.95) | 0.04 |
| Brain metastasis | Yes *v* no |  |  | 6.00(0.77-46.57) | 0.09 |
| Bladder metastasis | Yes *v* no | 4.90(1.07-22.4) | 0.04 | 2.24(1.09-4.59) | 0.03 |
| Metastatic organs | ≥2 *v* ＜2 | 4.09(1.13-14.73) | 0.03 | 2.88(1.41-5.90) | 0.004 |
| Immunohistochemical index[Syn] | Positive *v* negative | 1.18(0.14-9.59) | 0.88 | 0.88(0.31-2.55) | 0.82 |
| Immunohistochemical index[CgA] | Positive *v* negative | 1.10(0.28-4.27) | 0.89 | 0.82(0.41-1.65) | 0.57 |
| Gleason score | ≤9 *v ＞*9 |  |  | 1.01(1.00- 1.02) | 0.05 |
| Type of NEPC pathology | pure SCC *v* Adeno-SCC | 0.63(0.08-5.32) | 0.68 |  |  |
| Type of NEPC Treatment | CRT *v* surgery  CT *v* surgery | 3.05(0.46-20.27  )  1.76(0.29-10.84) | 0.25  0.54 |  |  |
| Type of NEDPC Treatment | CT *v* surgery  RT *v* surgery  CRT *v* surgery |  |  | 3.86(1.26-11.82)  11.03(2.57-47.35)  7.33(2.03-26.47) | 0.02  ＜0.001  0.002 |
| Abbreviations: NEPC, neuroendocrine prostate cancer; NEDPC, neuroendocrine differentiation prostate cancer; CgA, chromogranin A; Syn, synaptophysin; Pure SCC, pure small-cell carcinoma; Adeno-SCC, SCC admixed with adenocarcinoma; RT, radiotherapy; CRT, chemoradiotherapy; CT, chemotherapy. | | | | | |
